# Elevated serum levels of soluble CD14 in HBeAg‐positive chronic HBV patients upon Peginterferon treatment are associated with treatment response

**DOI:** 10.1111/jvh.13127

**Published:** 2019-06-03

**Authors:** Yingying Dou, Nadine van Montfoort, Aniek van den Bosch, Harry L. A. Janssen, Robert A. de Man, Sonja I. Buschow, Andrea M. Woltman

**Affiliations:** ^1^ Department of Gastroenterology and Hepatology Erasmus MC University Medical Center Rotterdam The Netherlands; ^2^ Institute of Medical Education Research Rotterdam Erasmus MC University Medical Center Rotterdam The Netherlands; ^3^ Present address: Department of Medical Oncology Leiden University Medical Center Leiden The Netherlands; ^4^ Present address: Toronto Center for Liver Disease, Toronto General Hospital University Health Network Toronto Canada

**Keywords:** clinical outcome, HBeAg+, HBV, PEG‐IFN, sCD14

## Abstract

Pegylated IFNα (PEG‐IFN) is one of the treatment options for chronic HBV (CHB) patients. However, the high patient treatment burden and limited response rate together clearly ask for biomarkers to predict PEG‐IFN response. Soluble CD14 (sCD14) is considered a marker for immune activation and has been shown to predict clinical outcome of HIV infection. However, studies on sCD14 in CHB infection are inconclusive, and its relationship with clinical outcome is largely unknown. Here, we measured sCD14 levels in CHB patients and investigated whether changes in sCD14 level related to PEG‐IFN response. Serum sCD14 levels were determined in 15 healthy controls, 15 acute self‐limited HBV, 60 CHB patients in different disease phases and 94 HBeAg+ CHB patients at week 0 and week 12 of a 52‐week PEG‐IFN treatment. Response to PEG‐IFN treatment was defined as HBeAg seroconversion or HBeAg loss at 26 weeks post‐treatment. The mean sCD14 level in acute HBV patients (3.0 µg/mL) was significantly higher than in CHB patients (2.4 µg/mL) and healthy controls (2.4 µg/mL). In CHB patients receiving PEG‐IFN, a significant increase in sCD14 was found after 12‐week treatment (median week 0:2.1 µg/mL; week 12:3.7 µg/mL). After 12‐week treatment, the fold change (FC = w12/w0) in sCD14 was significantly higher in responders compared to nonresponders (HBeAg seroconversion: median FC_responder_ = 2.1 vs FC_nonresponder_ = 1.6; HBeAg loss: median FC_responder_ = 2.2 vs FC_nonresponder_ = 1.5). Receiver operating characteristic curves demonstrated that FC‐sCD14_wk12/wk0_ levels can be of significant value as a stopping rule to select patients at week 12 who are not likely to benefit from further PEG‐IFN treatment.

**Abbreviations**
AUC: area under the ROC curve
CHB: chronic HBV
CV: Coefficients of variability
EASL: European Association for the Study of the Liver
HBV: hepatitis B virus
HCC: hepatocellular carcinoma
LAM: lamivudine
LPS: lipopolysaccharides
NA: nucleos(t)ide analogues
OD: Optical density
ROC: receiver operating characteristic

## 1 INTRODUCTION

About 1/3 of the world's population has been exposed to hepatitis B virus (HBV) during their lifetime, and over 250 million individuals worldwide suffer from chronic HBV (CHB) infection.^1^ These patients are at increased risk of developing liver fibrosis, cirrhosis and hepatocellular carcinoma (HCC).^2^ Currently, there are two main treatment options for CHB patients: nucleos(t)ide analogues (NA) and pegylated IFNα (PEG‐IFN). NA interfere with viral replication but do not eliminate the virus have to be given lifelong and may lead to viral escape variants. The other treatment, PEG‐IFN, aims to induce long‐term immunological control over the virus after finite treatment duration, typically 1 year. PEG‐IFN, however, has a moderate antiviral effect resulting in a sustained response in only ~25% of patients^3^ and a serious risk of adverse events.^4^ Considering the limited response rate, high costs and severe side effects, indicators to predict which individuals are most likely to respond to PEG‐IFN, are highly needed. Especially, early on‐treatment predictors are of interest to discontinue PEG‐IFN and minimize treatment burden for the nonresponding patients.

CD14 is a protein that plays an important role in immunity. CD14 acts as a co‐receptor with Toll‐like receptor 4 for the detection of bacterial lipopolysaccharides (LPS).^5^ It also recognizes other pathogen‐associated molecular patterns such as bacterial lipoteichoic acid.^6^ It exists in two forms, a membrane‐attached form (mCD14) and a soluble form (sCD14). mCD14 is a glycosylphosphatidylinositol‐anchored glycoprotein expressed on the surface of various cells including myeloid cells^7^, ^8^ and nonhematopoietic liver cells.^7^ sCD14 is produced by activated monocytes, macrophages and primary human hepatocytes.^9^, ^10^ In serum, it may serve as a biological marker for inflammatory disease activity as increased serum levels of sCD14 have been detected in patients with bacterial infections, viral infections,^11^, ^12^ rheumatic diseases^13^, ^14^ and multiple sclerosis.^15^

sCD14 has been widely studied as a biomarker in HIV infection and liver disease. Elevated sCD14 levels were found in HIV‐infected patients compared with seronegative controls,^12^, ^16^, ^17^ and sCD14 levels could independently predict disease progression and mortality for both HIV‐1 and HIV‐2 infections.^16^, ^18^, ^19^ In viral hepatitis, high levels of baseline plasma sCD14 were associated with worse clinical outcome in a mixed cohort of CHB and chronic HCV patients.^20^ Furthermore, elevated serum sCD14 levels were associated with HBV‐related HCC in CHB.^21^ Finally in HCV, the rise in sCD14 after 12 weeks of PEG‐IFN treatment was a negative predictor of treatment response.^22^ For HBV, however, neither the relation between serum sCD14 levels and the natural history of disease nor clinical outcome upon PEG‐IFN treatment has been clearly established.

Here, we measured and analysed sCD14 levels in acute HBV infection, treatment‐naive CHB patients from various disease stages and HBeAg+ CHB patients receiving PEG‐IFN. For the latter, we characterized the relationship of sCD14 serum levels to PEG‐IFN treatment response and assessed the value of sCD14 as an early on‐treatment indicator to stop treatment of patients who are unlikely to respond.

## 2 MATERIALS AND METHODS

### 2.1 Patient selection

Sixty CHB patients who never received treatment attending the outpatient hepatology clinic of the ErasmusMC and 15 age‐matched, acute self‐limited HBV patients and healthy controls were included in our study (Table 1). Serum samples were prospectively collected and stored at −80°C. Exclusion criteria for selection in the study are described in detail elsewhere.^23^ A total of 94 HBeAg+ CHB patients, who were previously enrolled in an investigator‐initiated multicentre randomized trial (99‐01 study) comparing PEG‐IFN monotherapy or combination therapy with lamivudine (LAM), were also included.^24^ The inclusion and exclusion criteria are similar to the original study.^24^ Additional inclusion criteria for the current analysis were completion of the 26‐week follow‐up phase of the original study and availability of a baseline and 12‐week serum sample for quantification of sCD14 levels (n = 94). All patients gave written informed consent according to the standards of the local ethics committee.

**Table 1:** Patient characteristics

Characteristics	Healthy	Acute HBV	Treatment‐naive chronic HBV patients				PEG‐IFN treatment HBeAg‐positive CHB	
			Immune tolerant	Immune active	Inactive carrier	E‐negative		
No. of patients	15	15	15	15	15	15	94	
Age (y), Mean ± SD	35 ± 12	41 ± 13	31 ± 8	31 ± 9	42 ± 15	38 ± 10	34 ± 11	
Male (%)	6 (40%)	14 (93%)	3 (20%)	8 (53%)	5 (33%)	13 (87%)	77 (82%)	
Ethnicity								
Caucasian, n (%)	‐	‐	0 (0%)	1 (7%)	1 (7%)	2 (13%)	67 (71%)	
Asian, n (%)	‐	‐	14 (93%)	11 (73%)	7 (47%)	8 (53%)	19 (20%)	
Other, n (%)	‐	‐	1 (7%)	3 (20%)	7 (47%)	5 (33%)	8 (9%)	
Laboratory parameters								
ALT, *ULN, Median (IQR)	‐	9.7 (3.4, 15.6)	0.6 (0.5, 0.7)	1.7 (1.3, 2.4)	0.6 (0.5, 0.7)	1.3 (1.1, 2.1)	3.4 (2.3, 5.4)	
HBV DNA, log_10_ IU/mL, Median (IQR)	‐	4.2 (3.4, 4.9)	8.8 (8.4, 9.0)	7.7 (6.6, 8.8)	2.5 (2.0, 2.8)	4.1 (3.8, 5.6)	8.5 (7.9, 9.0)	
HBsAg, log_10_ IU/mL, Mean ± SD	‐	Positive	4.8 ± 0.2	4.3 ± 0.8	2.6 ± 1.1	3.5 ± 0.5	4.4 ± 0.7	
HBeAg, log_10_ IU/mL, Mean ± SD	‐	‐	3.0 ± 0.3	1.4 ± 1.5	‐	‐	2.4 ± 2.6	
HBeAg, positive (%)	‐	11 (73.3%)	15 (100%)	15 (100%)	0 (0%)	0 (0%)	94 (100%)	
HBV genotype								
A/B/C/D/other	‐	‐	‐	‐	‐	‐	26/10/16/38/4	
Treatment								
PEG‐IFN monotherapy	‐	‐	‐	‐	‐	‐	41 (44%)	
PEG‐IFN + Lamivudine(LAM)	‐	‐	‐	‐	‐	‐	53 (56%)	
Fibrosis (Fibroscan)							Fibrosis (Ishak score)	
F0, n (%)	‐	‐	3 (20%)	2 (13%)	3 (20%)	‐	0, n (%)	10 (11%)
F0‐F1, n (%)	‐	‐	11 (73%)	9 (60%)	11 (73%)	10 (67%)	1, n (%)	22 (23%)
F1, n (%)	‐	‐	‐	‐	‐	1 (7%)	2, n (%)	9 (10%)
F1‐F2, n (%)	‐	‐	‐	‐	‐	1 (7%)	3, n (%)	24 (26%)
F2, n (%)	‐	‐	‐	2 (13%)	‐	1 (7%)	4, n (%)	5 (5%)
F2‐F3, n (%)	‐	‐	‐	‐	‐	1 (7%)	5, n (%)	5 (5%)
F3, n (%)	‐	‐	‐	1 (7%)	‐	1 (7%)	6, n (%)	2 (2%)
Unknown, n (%)	‐	‐	1 (7%)	1 (7%)	1 (7%)	‐	Unknown, n (%)	17 (18%)

Note

Data are presented as mean ± SD, median (IQR) or n(%).

Abbreviations: ALT, alanine aminotransferase; ULN, upper limit of normal.

### 2.2 Study design

A total of 94 HBeAg‐positive CHB patients who are from 99‐01 study, as described before,^24^ in brief, received combination therapy with weekly doses of 100 μg PEG‐IFN (PegIntron, Schering‐Plough) and a daily dose of 100 mg LAM (Zeffix, GlaxoSmithKline) or monotherapy with 100 μg/wk PEG‐IFN and placebo. The total duration of treatment was 52 weeks. All the patients were followed up for 26 weeks post‐treatment. Patient serum samples that used for sCD14 measurement were collected at week 12 during PEG‐IFN treatment and stored at −80°C. To reduce the likelihood of early patient dropout, the dose of PEG‐IFN was lowered to 50 μg/wk in both treatment groups after 32 weeks.

### 2.3 Assessment of serum sCD14 levels and laboratory variables

Serum sCD14 was assessed by an in‐house‐developed ELISA as previously described.^25^ Coating antibody was purified rat anti‐human sCD14 (clone 55‐3, BD Biosciences). Detection antibody was biotinylated anti‐human sCD14 (clone 3‐C39, BD Biosciences). X‐Vivo medium (Invitrogen) was used for sample and standard dilution. Recombinant sCD14 from GeneTex (GTX48216‐PRO) was dissolved in PBS and used for standard curve. Assay diluent, used for blocking and dilution of antibodies, streptavidin‐HRP and TMB solution were all from eBioscience/Thermo Fisher. Wash buffer was PBS + 0.05% Tween‐20. Reaction was stopped with 1:3 v/v of H_2_SO_4_. Optical density (OD) at 450 nm was measured on a Bio‐Rad imager. Coefficients of variability (CV) of the ELISA were determined on 4 reference patient samples: inter‐assay CV is 7.4%, intra‐assay CV is 4.8%, detection range is 100 pg/mL‐0 ng/mL. All serum samples were 1000 times diluted. ELISA results were multiplied by 1000 to calculate sCD14 concentration in patient serum samples. Laboratory variables including ALT, HBsAg, HBeAg, HBV DNA, HBV genotype were measured based on the manufacturer's instructions as previously reported.^23^, ^24^

### 2.4 Statistical analysis

Data are presented as mean ± SD/SEM or median (interquartile range, IQR) or count (percentage) as appropriate. The Mann‐Whitney test, Wilcoxon signed‐rank test, *t* test were performed for comparisons between two independent groups as appropriate. Spearman's rank correlations were performed to evaluate the correlation between sCD14 with other parameters. HBV DNA, HBeAg and HBsAg levels were logarithmically transformed. To assess the change in sCD14 level, serum levels were first transformed with base 2 log to obtain a normal distribution and a fold change in sCD14 (FC‐sCD14) FC = 2^(log_2_^(sCD14week 12)^ ‐ log_2_^(sCD14week 0)^) value was calculated for each patient and used for statistical analysis. ANOVA and Tukey's multiple comparison test were performed for comparisons among multiple groups as appropriate. Two‐sided *P*‐values < 0.05 were considered statistically significant. The diagnostic performance of sCD14 was analysed by calculating the receiver operating characteristic (ROC) curve and area under the ROC curve (AUC). The sensitivity, specificity, positive predictive value and negative predictive value were calculated at the optimal cut‐off value. These analyses were performed using the GraphPad Prism 5 software (GraphPad Prism Software, Inc).

## 3 RESULTS

### 3.1 Patient characteristics

A total of 169 HBV patients were included in this study, of which 15 acute self‐limited HBV patients, 60 CHB patients who never received treatment from each of the four recognized clinical CHB phases (15 each) and 94 HBeAg+ CHB patients receiving PEG‐IFN treatment ± lamivudine (LAM). In addition, we included 15 age‐matched healthy controls. Patient characteristics are presented in Table 1. For patients receiving PEG‐IFN treatment, 82% (n = 77) were male. The mean age at baseline was 34 years. 56% of patients were receiving PEG‐IFN with LAM (n = 53), and 44% of patients were receiving PEG‐IFN only. 71% of patients were Caucasian (n = 67) and 20% Asian (n = 19). HBV genotypes A, B, C, D and other/mixed were present in 28% (n = 26), 11% (n = 10), 17% (n = 16), 40% (n = 38) and 4% (n = 4) of patients, respectively.

### 3.2 Acute but not chronic HBV infection or its disease phases associated with elevated sCD14 levels compared to healthy individuals

**Figure 1:**
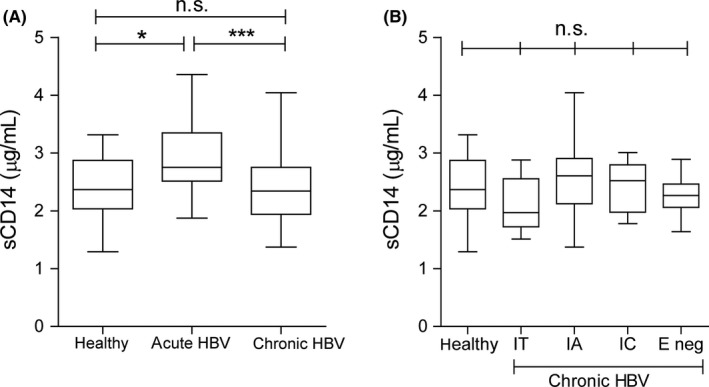
Serum sCD14 levels in healthy controls, acute HBV patients and chronic HBV patients. A, Box and whisker plots of serum sCD14 levels in healthy controls (n = 15), acute HBV patients (n = 15) and chronic HBV patients (n = 60). The top and bottom of the whiskers represent the measured maximum and minimum values, respectively. The bottom and top of the box represent the first and third quartiles, respectively. Mean sCD14 is depicted as horizontal lines within the boxes and were 3.0 µg/mL for acute HBV patients, 2.4 µg/mL for healthy controls and 2.4 µg/mL for chronic HBV (CHB) patients. *P < 0.05, ***P < 0.001 by one‐way ANOVA and Tukey's multiple comparison test. B, Box and whisker plots of serum sCD14 concentration of CHB patients as in A but stratified for the four phases of CHB infection. Means were 2.1 µg/mL for IT (Immune tolerant; n = 15), 2.6 µg/mL for IA (immune active; n = 15), 2.4 µg/mL for IC (inactive carrier; n = 15), and 2.3 µg/mL for E‐neg (HBeAg‐negative; n = 15). ns, not significant by one‐way ANOVA

In order to characterize serum sCD14 levels in different HBV disease phases, sCD14 levels were first assessed in an age‐matched cohort of acute HBV patients, treatment‐naive CHB patients and healthy controls. Serum sCD14 levels were significantly elevated in acute self‐limiting HBV patients (mean of 3.0 µg/mL) compared to CHB patients (2.4 µg/mL) or healthy control individuals (2.4 µg/mL) (Figure 1A). The sCD14 levels in acute HBV patients tended to positively correlate to serum ALT levels (Spearman *r* = 0.53, *P* = 0.05; data not shown). Next, we compared sCD14 levels among the four clinical CHB phases, categorized on virological, biochemical and serological parameters, but did not find any significant differences among these groups or between the groups and healthy controls (Figure 1B). Furthermore, for these CHB patients, we did not find any correlation between sCD14 and liver damage (by serum ALT) or fibrosis stage (data not shown).

### 3.3 sCD14 levels rise upon treatment with PEG‐IFN

The underlying mechanism of action of PEG‐IFN treatment is the activation of the immune system. To investigate the effect of this treatment on serum sCD14 levels and to assess association of sCD14 with clinical outcome, sCD14 levels were determined in 94 CHB patients from a retrospective cohort.^24^ sCD14 concentration in serum was assessed pretreatment (week 0) and at 12 weeks upon PEG‐IFN treatment. Patients in this cohort had been treated with PEG‐IFN ± LAM for 52 weeks, and the response to PEG‐IFN treatment was assessed at 26 weeks post‐treatment (week 78) as reported.^24^ Previously, it was determined that treatment of PEG‐IFN with LAM did not influence response rates compared with PEG‐IFN only,^24^ and we here did not detect a difference of sCD14 levels between CHB patients receiving PEG‐IFN with or without LAM (Table 2). Therefore, sCD14 data from these groups were pooled for further analysis (hereafter referred to as PEG‐IFN unless specified).

**Table 2:** Subgroup analysis of the serum sCD14 in the CHB patients receiving PEG‐IFN treatment

	N	sCD14 week 0 Median (IQR) μg/mL	*P*‐value	sCD14 week 12 Median (IQR) μg/mL	*P*‐value	Fold Change[Link] of CD14_(w12/w0)_ Median (IQR)	*P*‐value
Sex							
Male	77	2.2 (1.8, 3.1)	0.7	3.7 (2.8, 5.1)	0.6	1.6 (1.2, 2.1)	0.6
Female	17	2.0 (1.7, 2.8)		3.9 (2.7, 5.2)		1.7 (1.4, 2.7)	
Ethnicity							
Caucasian	67	2.3 (1.8, 3.3)	0.02	4.2 (3.4, 5.7)	<0.0001	1.7 (1.4, 2.3)	0.004
Asian	19	1.9 (1.3, 2.3)		2.6 (2.0, 2.8)		1.4 (1.0, 1.8)	
Other	8	1.9 (1.4, 2.9)		4.6 (3.6, 5.4)		2.3 (1.5, 2.9)	
HBV genotype							
A	26	2.3 (1.9, 3.1)	0.3	4.1 (3.6, 6.0)	0.02	1.8 (1.5, 2.6)	0.1
B	10	2.0 (1.3, 2.4)		2.7 (2.4, 5.0)		1.7 (1.1, 2.2)	
C	16	2.0 (1.8, 3.5)		3.0 (2.2, 3.9)		1.5 (1.1, 2.0)	
D	38	2.3 (1.8, 3.5)		3.8 (2.9, 5.5)		1.5 (1.2, 2.1)	
Other/mixed	4	1.7 (1.5, 3.6)		4.6 (2.5, 5.5)		2.0 (1.3, 2.8)	
Treatment							
PEG‐IFN monotherapy	53	2.0 (1.6, 3.0)	0.1	3.6 (2.6, 5.0)	0.4	1.6 (1.3, 2.4)	0.9
PEG‐IFN + LAM therapy	41	2.4 (1.9, 3.3)		4.0 (2.9, 5.2)		1.7 (1.3, 2.0)	
Response (HBeAg seroconversion)							
No	72	2.1 (1.8, 3.0)	0.8	3.7 (2.7, 4.6)	0.2	1.6 (1.3, 2.0)	0.049
Yes	22	2.0 (1.5, 3.3)		4.6 (3.2, 5.6)		2.1 (1.5, 2.7)	
Response (HBeAg loss)							
No	70	2.1 (1.8, 3.0)	0.4	3.6 (2.7, 4.7)	0.1	1.5 (1.3, 2.0)	0.01
Yes	24	2.0 (1.5, 3.0)		4.5 (3.4, 5.4)		2.2 (1.5, 2.7)	
All	94	2.1 (1.8, 3.0)		3.7 (2.8, 5.1)		1.6 (1.3, 2.2)	

Note

Mann‐Whitney test was used in sex, treatment and response analysis; one‐way ANOVA was used for genotype and ethnicity analysis.

Fold change of sCD14_(wk12/wk0)_ is calculated as described in Materials and Methods section.

**Figure 2:**
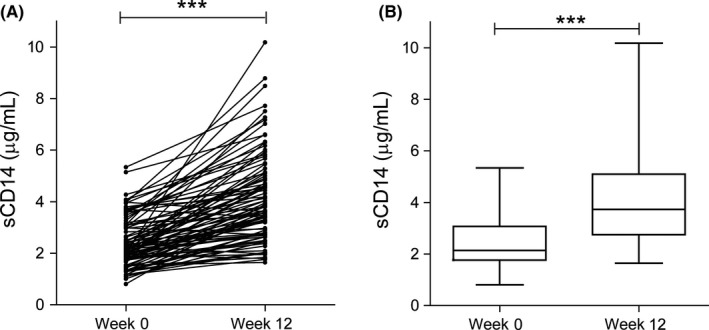
Elevated sCD14 levels in CHB patients upon treatment with PEG‐IFN. A, Serum level of sCD14 was measured at week 0 and at week 12 of PEG‐IFN treatment in 94 HBeAg+ CHB patients. Samples from one individual are connected by a line. B, Box and whisker plots of serum sCD14 for the data shown in A for better visualization of the distribution of levels. The top and bottom of the whiskers represent the measured maximum and minimum values, respectively. The bottom and top of the box represent the first and third quartiles, respectively. Medians (2.1 µg/mL at week 0, 3.7 µg/mL at week 12) are depicted within the box with the horizontal lines at indicated time points, n = 94. ***P < 0.0001 by Wilcoxon signed‐rank test

Baseline sCD14 levels did not significantly differ between sexes, or across HBV genotypes A‐D. Correlation between baseline sCD14 and baseline HBsAg level was observed (Spearman *r* = 0.22, *P* = 0.03; data not shown). Serum sCD14 levels were significantly elevated after 12 weeks of PEG‐IFN treatment in paired patient samples (Figure 2A). Median baseline level of sCD14 was 2.1 μg/mL (IQR 1.8‐3.0 μg/mL), and the median level at week 12 was increased to 3.7 μg/mL (IQR 2.8‐5.1 μg/mL) (Figure 2B). No correlation was found between sCD14 levels (week 0 and 12) and ALT, fibrosis (Ishak score) or necroinflammation. sCD14 level at week 12 was associated with genotype (Table 2). sCD14 levels were also associated with ethnicity; Asian patients in our cohort (n = 19) displayed significant lower sCD14 concentrations as compared to other ethnicities (Table 2), across all time points.

### 3.4 The change of sCD14 at week 12 of treatment is associated with treatment response

**Figure 3:**
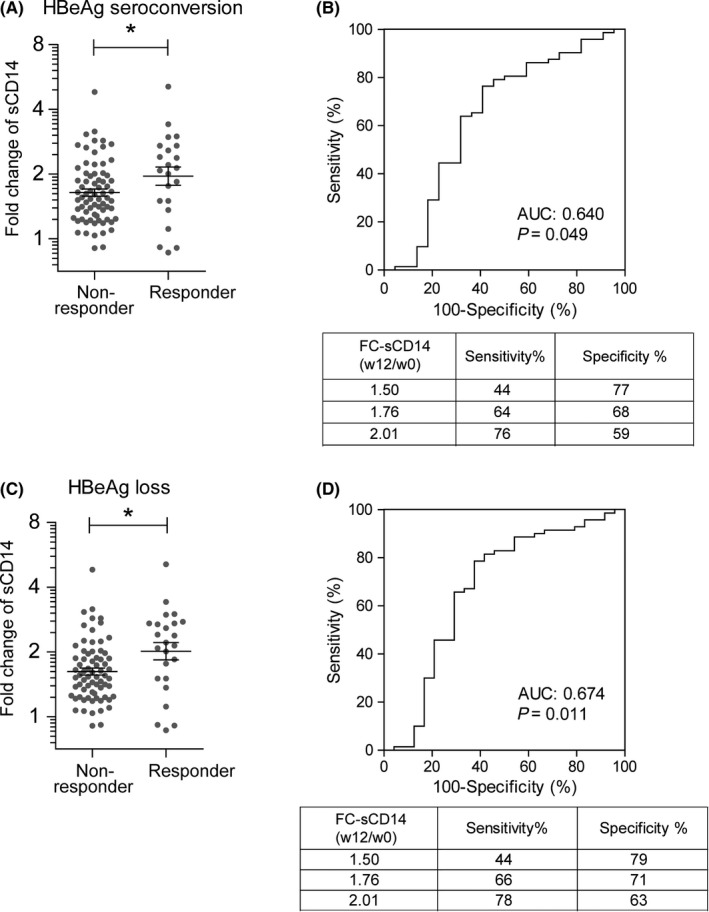
The change of sCD14 at week 12 of PEG‐IFN treatment is associated with clinical outcome. A, Individual values of FC‐sCD14_wk12/wk0_ and mean of responders (mean ± SEM = 2.0 ± 1.1) and nonresponders (1.6 ± 1.0) as defined by HBeAg seroconversion at week 78 are shown. Y‐axis is displayed in log scale with base 2. FC‐sCD14 is calculated as described in Materials and Methods section. Statistical analysis by Mann‐Whitney test: *P < 0.05. B, ROC curve and AUC selecting nonresponders based on the FC‐sCD14_wk12/wk0_ at week 12 depicted in panel A. As examples, sensitivities and specificities for the selection of nonresponders are provided for three FC‐sCD14 cut‐off values. C, Individual values and mean of FC‐sCD14_wk12/wk0_ of responders (2.0 ± 1.1) and nonresponders (1.6 ± 1.0) as defined by HBeAg loss at week 78. Y‐axis is displayed in log scale with base 2. Mann‐Whitney test on logarithmically transformed values. *P < 0.05. D, ROC curve and AUC selecting nonresponders using the FC‐sCD14_wk12/wk0_ at week 12 depicted in panel C. Sensitivities and specificities for three FC‐sCD14 cut‐off values are provided as examples

Next, we assessed the relation between sCD14 levels and clinical outcome of PEG‐IFN treatment. Response to PEG‐IFN treatment was determined at 26 weeks post‐treatment (week 78) and defined as HBeAg seroconversion or HBeAg loss. Overall, 22 patients (23.4%) displayed HBeAg seroconversion, 24 patients (25.5%) showed HBeAg loss, and 22 patients (23.4%) exhibited both HBeAg loss and HBeAg seroconversion (Figure S1A). After 12 weeks of treatment, the median sCD14 level was significantly increased for both responders and nonresponders defined by either HBeAg seroconversion or HBeAg loss (Figure S1B,C). Neither pretreatment nor week 12 sCD14 levels correlated with PEG‐IFN treatment response regardless of how it was defined (Table 2). Interestingly, we found that the mean FC‐sCD14_(wk12/wk0)_ was significantly higher in responders compared to nonresponders (FC_responder_ = 2.0 vs FC_nonresponder_ = 1.6 for both HBeAg seroconversion and HBeAg loss) (Figure 3A‐D). Receiver operating characteristic (ROC) curves and area under the curve (AUC) values demonstrated that the FC‐sCD14_(wk12/wk0)_ can be used to select patients at week 12 that likely do not benefit from further PEG‐IFN treatment (Figure 3B,D). For better appreciation of the predictive value of FC‐sCD14_(wk12/wk0)_, we provided sensitivities and specificities for three cut‐off values (FCs 1.50, 1.76 and 2.01) for each response definition (Figure 3B,D). Importantly, FC‐sCD14_(wk12/wk0)_ was not associated with sex, HBV genotypes and treatment, however, was influenced by ethnicity as was also observed for the sCD14 levels at baseline and week 12 (Table 2). Because ethnicity is a cofounder for FC‐sCD14_(wk12/wk0)_ levels, we also performed this operation without the Asian patients (Figure S2). When excluding Asian patients, the AUC was even higher (Figure S2).

Knowing FC‐sCD14_wk12/wk0_ is associated with HBeAg seroconversion and HBeAg loss, we next studied the correlation of FC‐sCD14 with the change in viral parameters. For this, an FC‐sCD14_(wk12/wk0)_ cut‐off of 1.5 or 2.0 was used to divide patients into two groups for which we calculated on‐treatment changes in HBV DNA, HBeAg and ALT (Figure S3A,B). All parameters declined profoundly towards week 52 but increased again post‐treatment (week 78). CHB patients with a high change in FC‐sCD14_wk12/wk0_ (>2.0) displayed a significant decline in HBV DNA, HBeAg and ALT after PEG‐IFN treatment at week 78. This was not the case when groups were formed using the lower FC cut‐off of 1.5 and was not at all observed for HBsAg. No significant correlations were found between either sCD14 levels (pretreatment & at week 12) or the FC‐sCD14_wk12/wk0_ and changes in HBV DNA, HBeAg, HBsAg and ALT post‐treatment (week 78; data not shown).

### 3.5 Predictive value of the change of sCD14 at week 12 upon PEG‐IFN treatment

For the treatment of CHB patients in European countries, the European Association for the Study of the Liver (EASL) guideline is the general reference. The 2017 EASL guideline states “In HBeAg+ CHB patients, HBsAg levels >20,000 IU/ml for genotype B and C, or no decline of HBsAg levels for genotype A and D, at 12 weeks of PEG‐IFN therapy are associated with a very low probability of subsequent HBeAg seroconversion and can be used as PEG‐IFN stopping rules”.^26^ Using this EASL stopping rule, we retrospectively analysed our CHB patient cohort again, but now excluding patients who were not HBV genotype A‐D to match the EASL guideline that only applies to these genotypes. We found only 36% nonresponders can be excluded at week 12 upon PEG‐IFN treatment (specificity 80%; accuracy: 46%, 95% CI: 0.35‐0.56), leaving 64% nonresponders to undergo ineffective yet invasive PEG‐IFN treatment. However, by using FC‐sCD14_wk12/wk0_ at a cut‐off value of 1.5, we reached a higher sensitivity (43%) and the same specificity (80%) as when applying EASL HBsAg‐based stopping rules to exclude nonresponders. When using an FC‐sCD14_wk12/wk0_ cut‐off value of 2.0, even 76% nonresponders could be selected at week 12, with an accuracy of 72% to classify patients correctly (95% CI of accuracy: 0.62‐0.81, Table 3).

**Table 3:** Predictive value of the change of sCD14 at week 12 upon PEG‐IFN treatment

Week 12	2017 EASL guideline		Fold change of sCD14 cut‐off			
	Based on HBsAg level[Link]		2.0		1.5	
	Value	95% CI	Value	95% CI	Value	95% CI
Sensitivity (true nonresponder rate)	36%	0.25‐0.48	76%	0.64‐0.85	43%	0.31‐0.55
Specificity (true responder rate)	80%	0.56‐0.94	60%	0.36‐0.81	80%	0.56‐0.94
Positive predictive value	86%	0.71‐0.94	87%	0.79‐0.92	88%	0.75‐0.95
Negative predictive value	26%	0.21‐0.32	41%	0.29‐0.55	29%	0.23‐0.35
Accuracy	46%	0.35‐0.56	72%	0.62‐0.81	51%	0.40‐0.62

Note

90 CHB patients of genotype A‐D receiving PEG‐IFN were included in this analysis.

Responder to PEG‐IFN treatment was defined as HBeAg seroconversion at week 78.

Fold change of sCD14_(wk12/wk0)_ is calculated as described in Materials and Methods section.

In HBeAg+ CHB patients (26), at week 12: HBsAg > 20 000 IU/mL for genotype B & C or no decline of HBsAg for genotype A & D.

Combining of both markers revealed an even higher sensitivity (84% for FC‐sCD14_wk12/wk0_ = 2.0 and 61% for FC‐sCD14_wk12/wk0_ = 1.5 in combination with HBsAg) when compared with using either HBsAg or for FC‐sCD14_wk12/wk0_ alone (Table S1 and Table 3). Thus, it may be possible to exclude even more nonresponders after 12 weeks of treatment when using both serological markers together.

Taken all together, like HBsAg decline, the change in sCD14_wk12/wk0_ may thus also serve as an effective stopping criterion and may even be a superior negative predictive value.

## 4 DISCUSSION

This is the first study to describe the association of sCD14 levels with PEG‐IFN treatment response in CHB. We found that a high rise in serum sCD14 in CHB patients at the first 12 weeks of PEG‐IFN treatment is associated with an increased probability of clinical response defined as either HBeAg seroconversion or HBeAg loss. Besides this major finding, we also found serum sCD14 levels elevated in acute self‐limited HBV but not in CHB relative to healthy controls. In addition to the HBsAg‐based stopping rule provided in the 2017 EASL guideline for PEG‐IFN treatment for CHB, our results indicate that sCD14 may serve as an additional predictive marker to determine the probability of sustained response to PEG‐IFN in HBeAg+ CHB patients at week 12.

So far, only limited studies on sCD14 in CHB infection were reported. Similar to our observation, Oesterreicher et al^27^ found elevated sCD14 serum levels in acute but less in chronic viral hepatitis compared to healthy controls. This study, however, included a mixed population of acute hepatitis (HAV and HBV) and chronic hepatitis (HBV and HCV) patients of limited size. Steyaert et al^28^ in contrast did not find any elevation of sCD14 in acute HBV compared to healthy controls, while levels were higher in CHB patients. Similarly, also in studies by Li et al^21^ and Sandler et al,^20^ CHB patients were investigated and sCD14 levels were found to be elevated in both studies, which related to fibrosis and hepatic inflammation. Also, the latter study, however, was based on a mixed cohort containing CHB and HCV patients who were not analysed separately. The discrepancies among these studies and our own data may be caused by differences in the patient populations, level of fibrosis and/ or the sampling moment in case of acute HBV infection.^27^

Differences in patient baseline characteristics are often a barrier for direct comparison of data obtained from different studies. In our cross‐sectional study, treatment‐naïve CHB patients were categorized into four clinical phases^29^ based on virological, biochemical and serological parameters of equal numbers of patients in each disease phase with a similar age group. We observed that the mean sCD14 level in the immune active phase of CHB was slightly higher as compared to the mean sCD14 level found in healthy controls which is in line with the previously observed relation between hepatic inflammation and sCD14 levels.^20^ Possibly, previous studies reporting increased sCD14 levels in CHB included a subset of CHB patients with more severe liver disease,^20^, ^21^ while in our cohort, ~86% treatment‐naive CHB patients have a relative low fibrosis level (<F2 by fibroscan). Furthermore, we here demonstrate that ethnicity influences sCD14 levels which was reported before in rheumatoid arthritis patients^30^ and we previously reported the same for age,^25^ indicating that also these parameters may be a cause of discrepancy.

Why and how sCD14 is secreted remains elusive. sCD14 is thought to be produced by monocytes, macrophage, Kupffer cells and primary human hepatocytes, which can be increased upon activation by, for example, pathogen‐associated patterns and/or pro‐inflammatory cytokines. The pro‐inflammatory cytokine PEG‐IFN is a very potent activator of the immune system^31^, ^32^ that could directly or indirectly enhance release of sCD14 by hepatocytes and/or myeloid cells.^7^, ^8^, ^33^ In our study, we found that the elevation in sCD14 in responders to PEG‐IFN was significantly higher compared with those in nonresponders, which could therefore mean that inflammation/immune activation may be more effective and/or different in responders. The PEG‐IFN induced increase in sCD14 fits well with the notion that sCD14 is a marker of immune activation in a number of acute and chronic inflammatory conditions, including liver inflammation.^30^, ^34^ Since microbial translocation is known to contribute to inflammation in viral hepatitis patients,^20^ the observed increased sCD14 levels in HBV patients might also be partly due to release of CD14 from Kupffer cells upon stimulation by microbial products such as LPS.^5^

We previously demonstrated that HBsAg can activate Kupffer cells.^35^ In addition, we showed that HBsAg‐sCD14 complexes exist in serum of CHB patients and that HBsAg induces dendritic cell activation in a CD14‐dependent manner.^25^ Preliminary experiments suggest that PEG‐IFN stimulation may trigger sCD14 release by cultured Kupffer cells especially when HBsAg is present (unpublished data). Whether this may explain the correlation between HBsAg levels and sCD14 levels prior PEG‐IFN treatment, as demonstrated in our study remains to be elucidated. However, others reported an inverse correlation between plasma sCD14 and HBsAg concentrations in CHB patients,^28^ which emphasizes the need for further research to unravel the underlying mechanisms and the possible role and relation of sCD14 with HBsAg.

PEG‐IFN has been shown to be effective, that is, achieving immunological control over HBV also after ending of treatment, only in a minority of CHB patients. However, the limited number of patients who respond together with the high costs and side effects makes it essential to select patients who will not benefit from this therapy as early as possible.^36^ Previously, many studies have searched for response predictors at baseline. HBV genotype, baseline ALT, baseline HBV DNA,^36^, ^37^ fibrosis,^38^ core/precore mutations^39^ and IP‐10 levels^40^ were all reported as pretreatment predictors of PEG‐IFN response. Buster et al^37^ constructed a baseline prediction model allowing prediction of sustained response to PEG‐IFN for CHB patients with a high likelihood of response (https://liver-gi.nl/hepatitis-b). Yet, we did not find any relationship of sCD14 levels at week 0 or the change of sCD14 at week 12 with these reported pretreatment predictors in our cohort.

Despite these baseline predictors of PEG‐IFN response, on‐treatment predictors of PEG‐IFN response have also been studied. Monitoring HBV DNA^41^ levels during treatment to predict response has limited benefits. Serum HBeAg level during treatment had been reported inadequately to predict treatment response in HBeAg+ CHB patients.^39^, ^42^ Serum HBsAg upon treatment at week 12 combined with HBV genotype has been shown to be associated with HBeAg seroconversion at 6 months post‐treatment,^43^ and this HBsAg‐based stopping rule is included later in 2017 EASL guidelines.^26^ Recently, some new on‐treatment predictors like hepatitis B core‐related antigen (HBcrAg) and HBV RNA have been studied on the PEG‐IFN treatment response (review by Ref.^44^); however, further study is required to validate the clinical impact of these markers.

The 2017 EASL guidelines recommend to combine HBV genotype and HBsAg levels to predict the probability of subsequent HBeAg seroconversion and to stop the PEG‐IFN therapy at week 12 or week 24 for HBeAg+ CHB patients.^26^ Our study shows that the response to PEG‐IFN therapy in HBeAg+ patients can also be deduced from the change of serum sCD14 at week 12 with respect to week 0. Selecting nonresponders based on the change in sCD14 levels was even slightly more sensitive than the stopping rules defined in the 2017 EASL guideline. Importantly, the association of sCD14 change with response to treatment was independent of HBV genotype which makes it unique compared to other reported predictors so far. The value of using combinational markers of sCD14 change and HBsAg level for treatment response should be validated by others in future. These results demonstrate that sCD14 could serve as a valuable additional early immune marker for PEG‐IFN treatment response to stop ineffective treatment early on.

Although this study is the first to describe that the change of sCD14 might be used as an stopping rule in PEG‐IFN treatment at week 12, there are some limitations. First, a relatively small number of patients were included in our study that limited us to draw firm conclusions on the use of sCD14 as a biomarker in CHB treatment. Second, although the original study contained 2 treatment arms, we decided to pool the patients since LAM did not add to overall response rate,^24^ and no differences in sCD14 levels were found between CHB patients receiving PEG‐IFN with/without LAM (Table 2). Third, we were not able to perform external validation on using the change in sCD14 as an stopping reference for PEG‐IFN treatment at week 12, because an additional independent data set is not available to us. Thus, follow‐up studies on other patient cohort are needed to validate the use of sCD14 to stop PEG‐IFN treatment for those who will not benefit. Fourth, ethnicity is a confounder in our study. Our data suggest that predictive value of sCD14 may be lower in patients with Asian ethnicity that had lower sCD14 concentrations across all time points. Since only a limited number of patients with Asian ethnicity were represented in our cohort, validation of our findings in an independent cohort with more Asian patients is needed.

In conclusion, serum sCD14 has the potential as a biomarker to select patients who are unlikely to respond to PEG‐IFN treatment and thus suffer unnecessarily from its severe side effects. Our data indicate that a high rise in serum sCD14 level upon treatment is associated with a successful HBV‐directed immune response. This finding contributes to our understanding of the immunological requirements for viral eradication and calls for further research.

## CONFLICT OF INTEREST

The authors declare that there is no conflict of interest regarding the publication of this article.

## AUTHORS’ CONTRIBUTIONS

YD, NM, SIB, AMW and HJ designed the study. YD, AB and NM performed the experiments and analyses. YD and AMW wrote the manuscript. SIB and AMW supervised the study. NM, HJ, RAM, SIB and AMW critically reviewed the manuscript.
