# Supplementary material for: Elevated serum levels of soluble CD14 in HBeAg‐positive chronic HBV patients upon Peginterferon treatment are associated with treatment response
Source: J Viral Hepat. 2019 Jun 3;26(9):1076–85. doi: 10.1111/jvh.13127 (PMC6852593; doi:10.1111/jvh.13127)
Supplement: Supplementary file 4 [file JVH-26-1076-s004.docx]

| **Week12** | **2017 EASL guideline**^§^ **& Fold change of sCD14 cut-off** | | | |
| --- | --- | --- | --- | --- |
|  | 2 | | 1.5 | |
|  | Value | 95% CI | Value | 95% CI |
| **Sensitivity** | 84% | 0.73-0.92 | 61% | 0.49-0.73 |
| **(true non-responder rate)** |  |  |  |  |
| **Specificity** | 45% | 0.23-0.68 | 60% | 0.36-0.80 |
| **(true responder rate)** |  |  |  |  |
| **Positive predictive value** | 84% | 0.78-0.89 | 84% | 0.75-0.90 |
| **Negative predictive value** | 45% | 0.28-0.63 | 30% | 0.22-0.41 |
| **Accuracy** | 76% | 0.65-0.84 | 61% | 0.50-0.71 |

Supplementary Table 1. Predictive value of the change of sCD14 in combination with HBsAg at week12 upon PEG-IFN treatment.

^§^ In HBeAg+ CHB patients (23), at week 12: HBsAg > 20,000 IU/ml for genotype B & C or no decline of HBsAg for genotype A & D.
90 CHB patients of genotype A-D receiving PEG-IFN were included in this analysis.
Responder to PEG-IFN treatment was defined as HBeAg-seroconversion at week 78.
Fold-change of sCD14_(wk12/wk0)_ is calculated as described in Materials and Methods section.
